# Effects of Hydrotherapy on the Management of Childbirth and Its Outcomes—A Retrospective Cohort Study

**DOI:** 10.3390/nursrep14020095

**Published:** 2024-05-20

**Authors:** Elena Mellado-García, Lourdes Díaz-Rodríguez, Jonathan Cortés-Martín, Juan Carlos Sánchez-García, Beatriz Piqueras-Sola, Juan Carlos Higuero Macías, Raquel Rodríguez-Blanque

**Affiliations:** 1 Department of Nursing, Faculty of Health Sciences, University of Granada, 18016 Granada, Spain; e.elenamellado@go.ugr.es (E.M.-G.); cldiaz@ugr.es (L.D.-R.); jcortesmartin@ugr.es (J.C.-M.); rarobladoc@ugr.es (R.R.-B.); 2 Virgen de las Nieves University Hospital, 18014 Granada, Spain; bpiquerassola@gmail.com; 3 Costa del Sol University Hospital, 29603 Marbella, Spain; juancarlos.higueromacas@gmail.com; 4 San Cecilio University Hospital, 18016 Granada, Spain

**Keywords:** hydrotherapy, waterbirth, immersion, first labor stage, maternal health

## Abstract

The use of hydrotherapy during childbirth has gained relevance due to the demand for natural childbirth and greater respect for the woman’s choice. Studies have shown benefits such as less use of epidural analgesia, increased ability to cope with pain, shorter labor, and a better overall birth experience. Objective: The main objective of this study was to generate further evidence on maternal and birth outcomes associated with the use of hydrotherapy during labor, specifically aiming to describe the effects of water immersion during all stages of labor (first, second, and third) on women. Methodology: A retrospective cohort study was carried out on a random sample of women who gave birth at the Costa del Sol Hospital between January 2010 and December 2020. The calculated sample size was 377 women and the data were extracted from their partograms. After data extraction, two groups were formed: one group used hydrotherapy during childbirth (*n* = 124), while the other group included women who did not use hydrotherapy during the childbirth process (*n* = 253). Results: The results highlight significant differences in pain perception, analgesia use, types of labor, and delivery times between the two groups. Women who did not use hydrotherapy reported higher pain perception, with a median (IQR) of 8 (7–9) on a numerical scale, compared to a median (IQR) of 6 (5–7) for the hydrotherapy group. Furthermore, the group without hydrotherapy required epidural analgesia in 40% of cases, while in the hydrotherapy group, it was only necessary in 20%. In terms of the type of delivery, the hydrotherapy group had more spontaneous vaginal deliveries compared to the non-hydrotherapy group, which had more operative vaginal deliveries. The overall duration of labor was longer in the hydrotherapy group, especially in women who arrived at the hospital late in labor. Conclusions: Hydrotherapy is associated with a longer time to delivery. Women with a higher pain tolerance tend to opt for hydrotherapy instead of epidural analgesia.

## 1. Introduction

Medical procedures and care during childbirth are more popular than ever. It is necessary to personalize the care and resources available for childbirth, considering the pain, situation, and individual desires of each woman. Among the available non-pharmacological resources is hydrotherapy, which can be combined with relaxation and psychological techniques.

Hydrotherapy during childbirth offers benefits such as buoyancy and temperature regulation, supported by studies [1,2,3,4,5]. It is a mother-centered method for pregnancy and childbirth, providing comfort and utility [6]. Additionally, hydrotherapy improves the ability to have a natural and physiological birth, enhancing the overall childbirth experience [5].

Among the effects of hydrotherapy are a reduction in the use of epidural analgesia [4,7,8,9,10,11]; an improvement in the ability to cope with pain [3,7,12]; shorter labor [8,10,11,13,14]; an increased sense of control, according to some authors’ studies [1,15]; and positive effects on maternal [4] and neonatal [16] health. The Clinical Practice Guideline on Normal Childbirth Care in Spain [17] recommends immersion in hot water as an effective method for relieving pain during the final stage of childbirth.

The main objective of this study was to evaluate the effect of hydrotherapy during childbirth, specifically on delivery times.

## 2. Materials and Methods

### 2.1. Study Design

This was a retrospective cohort study of women who elected to receive hydrotherapy during their labor. The report of this research adheres to STROBE reporting guidelines for observational research [18].

### 2.2. Setting

Women who gave birth at the Costa del Sol Hospital, Málaga (Spain) during the period from January 2010, when the use of hydrotherapy during childbirth began to be offered at the hospital, to December 2020 were included in the study. In this hospital, the use of hydrotherapy is indicated for use in the first stage of labor. Exceptionally, if the woman refuses to leave the water and proceeds to the second stage of labor in water, her decision is respected, but this is not the hospital’s offering nor the objective of this study.

The data were collected from the partogram of each woman, as well as from the medical records of both the woman and the newborn.

### 2.3. Participants

In our study, we included women with a low-risk pregnancy and birth, meaning those with a healthy singleton pregnancy, a body mass index ≤ 30 kg/m^2^, cephalic presentation, spontaneous onset of labor, a gestational age between 37 + 0 and 41 + 6 weeks, and a normal cardiotocographic registry at admission. Women with twin pregnancies were excluded, as well as those with pre-term (<37 weeks) or post-term (>42 weeks) delivery.

Per protocol of our Birthing Unit, all women admitted to the unit are offered the possibility of using hydrotherapy during the delivery process.

### 2.4. Variables and Data Sources

The study meticulously planned data encoding in advance, directly extracting from medical records into a structured database. Sociodemographic and anthropometric variables, alongside the utilization of hydrotherapy during labor, were partitioned into two distinct tables for analysis. The principal variables encompassed age, parity, and labor duration, sourced from both clinical history records and the partograms. Additional recorded variables encompassed the type and onset of labor, analgesia and oxytocin administration, labor pain, and occurrences such as membrane rupture and the third stage of labor being recorded.

### 2.5. Bias

The study addressed biases by establishing clear inclusion and exclusion criteria for its participants, ensuring data anonymity, and conducting meticulous data encoding. Additionally, confounding variables were controlled through multivariable statistical analysis. These measures ensured the validity and reliability of the findings obtained in this retrospective cohort study.

### 2.6. Study Size

Referring to the systematic review by Cluett et al. [19], for the primary objective of this study, i.e., to compare the durations of their first stage of labor, a time less than 16 min between the hydrotherapy group and the non-hydrotherapy group was considered statistically relevant. For a known standard deviation of 48 min for each group (referring to the 2010 Torkamani, Kangani, and Janani study [20]), a type I error (alpha) of 0.05, and a type II error (beta) of 0.20, 111 patients were required per group. Estimating a loss of 10% in the evaluation of medical records, the total number of patients to be evaluated was 248 (124 patients per group).

The calculated sample size for this study was 248 pregnant women, including a control group of 124 women and 124 women in the hydrotherapy group.

To reach a sample size of 124 women in the hydrotherapy group, the sample was expanded to a total of 377 women, distributed among 253 individuals in the control group and 124 in the experimental hydrotherapy group.

### 2.7. Statistical Methods

Descriptive analyses were conducted using measures of the central tendency and dispersion (mean and standard deviation) for quantitative variables, and the frequency distribution for qualitative variables. To evaluate differences between the study groups (no hydrotherapy use vs. use of hydrotherapy), the chi-square test (or Fisher’s exact test in case of expected frequencies less than 5) was used for the categorical variables, and the Student’s *t*-test was used for the quantitative variables. Using pain as the outcome variable, a multivariate linear regression model was employed, including unbalanced independent variables from the previous bivariate analysis, selecting variables with a criterion of *p* < 0.05, and describing the beta coefficient (β) with respective 95% confidence intervals (CI95%). This involved checking for normality, homoscedasticity, and multicollinearity.

For all analyses, the level of statistical significance was set at *p* < 0.05. These analyses were performed using SPSS vs. 28.0 program for Windows (IBM Corporation, Armonk, NY, USA) statistical software.

### 2.8. Ethics Statement

This study was conducted in accordance with the Declaration of Helsinki for research involving human subjects. The Ethics Committee of the Costa del Sol Hospital approved this study in November 2018 under reference number 002_oct18_PI-hydrotherapy birth, thus ensuring the ethical compliance of the research in question.

No personal or identifying information was collected. Anonymity was guaranteed thanks to the research service of the Costa del Sol Hospital, which anonymized the personal or identifying data of the women involved in the study. In addition, the data were stored in a password-protected personal computer.

## 3. Results

Data were collected from a total of 377 women (*n* = 253 for those who did not use hydrotherapy; *n* = 124 for those who used water during labor). There were no statistically significant differences in their age (*p* = 0.103; non-hydrotherapy group: 32.4 ± 5.3 years, and hydrotherapy group: 31.7 ± 5.6 years).

Table 1 shows the baseline characteristics of the sample.

The use of hydrotherapy is influenced by the profile of the parturient; it is observed that there are statistically significant differences between women presenting their first pregnancy and those who presented their second or third gestation onwards (chi-square of 12.153 with a significance of 0.002). Among the women who chose to use hydrotherapy, women with no previous experience in childbirth were more likely to use hydrotherapy (43.4%) than women who had had a previous birth (28.8%) and those who had had more than one previous birth (23.5%). Related to this is the number of previous children, which is significant for the women with no previous children versus those with one or more than one (*p* < 0.001).

The presence of previous miscarriages does not differ significantly between the groups and did not influence the women’s requests for hydrotherapy or no hydrotherapy (*p* = 0.6). Conventional management of labor, which includes the use of oxytocin and artificial rupture of membranes, was occasionally employed. The hydrotherapy group exhibited lower oxytocin usage (8.9%), and concerning the artificial rupture of membranes during labor, there was more oxytocin administration in the non-hydrotherapy group (8.1%) compared to the hydrotherapy group. Thus, fewer uses of oxytocin and artificial ruptures of membranes were observed in the hydrotherapy group, with significant differences being noted between the two groups regarding oxytocin administration (*p* = 0.007).

Regarding the types of delivery, the highest percentage was concentrated in “Spontaneous vaginal delivery”, accounting for 94.7% of the total women. Those who did not use hydrotherapy represented 93.3%, while those who did represented 97.6%. For “Operative vaginal delivery”, it was observed that “ventouse” was used in 3.2% of women who did not use hydrotherapy, compared to 1.6% of those who did. Forceps were used for 1.2% of the women who did not use hydrotherapy, and no use of these assisted techniques was observed in the women who used hydrotherapy. Regarding “Cesarean delivery”, the percentage was 2% in the women who did not use hydrotherapy, compared to 0.8% in those who did.

It is observed that hydrotherapy, with a mean of 6 ± 1, is associated with a lower pain perception compared to the absence of hydrotherapy, which presented a mean of 8 ± 1.

**Figure 1:**
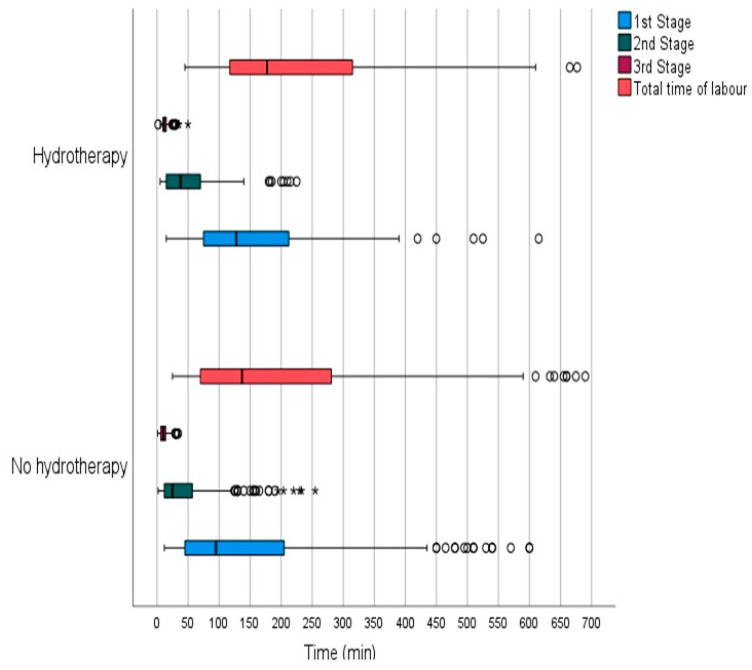
Median durations of labor considering labor stages and hydrotherapy use. Circle represents mild outlier, asterisk represents extreme outlier.

Many of these women opted for the use of epidural analgesia at some point during labor. The utilization of epidural analgesia in the hydrotherapy group was 20.2%, while in the non-hydrotherapy group, 40.7% of the women demanded epidural analgesia, with the difference being statistically significant (*p* < 0.001). The median duration of labor was longer for the women who utilized hydrotherapy during labor compared to those who did not (140 min vs. 180 min; *p* = 0.002), as illustrated graphically in Figure 1.

The median duration of the first stage of labor (dilation) was 105 min for the group that did not use hydrotherapy compared to 130 min for the group that used hydrotherapy (*p* = 0.007). The median duration of the second stage of labor (expulsion) was 40 min for women who used hydrotherapy and 25 min for those who did not use hydrotherapy (*p* = 0.021). Delivery times in the third stage of labor showed significant differences (*p* = 0.002), with better results for women who did not use hydrotherapy compared to those who did.

**Figure 2:**
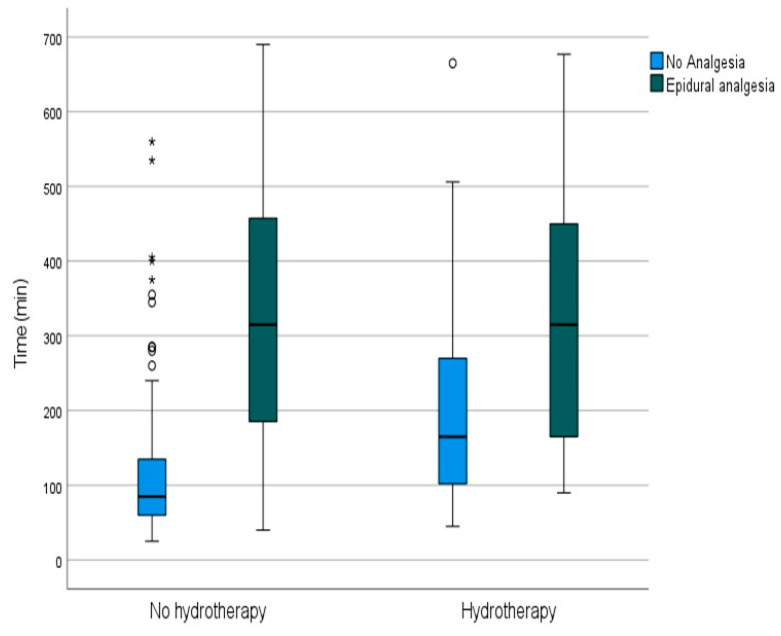
Mean durations of labor considering hydrotherapy use and analgesia use. Circle represents mild outlier, asterisk represents extreme outlier.

Referring to the results obtained for both the use of epidural analgesia and the delivery times, and taking into account the aforementioned results with respect to the overall delivery times, we compared the group that used analgesia and did not use hydrotherapy with the group that used analgesia and did use hydrotherapy; the differences were not significant (Figure 2).

**Figure 3:**
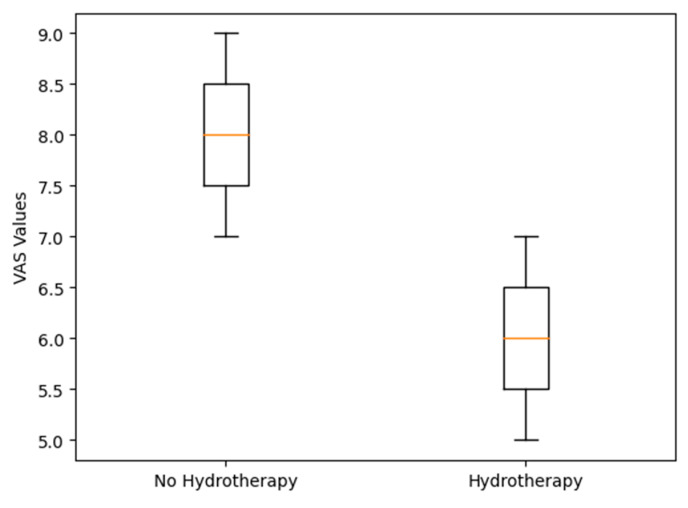
Distribution of VAS values considering the use of hydrotherapy.

The women in our study experienced greater pain relief during labor with the use of hydrotherapy. The differences were significant (*p* < 0.001) between the two groups: the group that did not use hydrotherapy presented a mean value of 8 on the numerical pain scale compared to a value of 6 in the group that used hydrotherapy (Figure 3).

In the multivariate linear regression model, in which the variables previous gestation, age, and use of hydrotherapy were related to pain during labor, we found that the β value indicated that the presence of hydrotherapy reduced pain by a score of 1.56 (95% CI (1.26–1.86)) compared to the non-hydrotherapy group scores, adjusted for both age and previous gestation.

## 4. Discussion

Our study revealed statistically significant differences in the duration of different stages of labor between women who used hydrotherapy and those who did not. Specifically, we observed a shorter first stage of labor among women who utilized hydrotherapy compared to those who did not (*p* = 0.007; median 130 min (IQR = 153.75 min) vs. median 105 min (IQR = 165 min)), while the duration of the second stage of labor was comparable between the two groups (*p* = 0.021; median 40 min (IQR 57.25 min) vs. median 25 min (IQR 47.5 min)). Significant differences were also found in the third stage of labor (*p* < 0.000), favoring those who did not use hydrotherapy (median 10 min (IQR 8.5 min) vs. median 10 min (IQR = 5 min)). It is noted that the disparity in labor durations may be attributed to differences in the composition of the population groups studied, especially regarding the parity of the women.

Our findings contrast with those presented in the studies by Schorn et al. [21] and Cluett et al. [22], who found no significant differences in the total duration of labor or in the duration of the first stage of labor between women who utilized hydrotherapy and those who did not. Conversely, the results of the study by Chaichian et al. [14] demonstrate significant differences in the duration of the active phases of the first and third stages of labor between groups of women who used hydrotherapy and those who did not. This is consistent with our findings, as we also observed significant differences in the duration of the first stage of labor between the two groups of women.

Our study highlights the impact of hydrotherapy on pain during labor. We found that its use was associated with less pain, with a mean score of 6 on the pain visual analog scale, compared to a mean score of 8 in women who did not use hydrotherapy (*p* < 0.001). Additionally, women who did not use hydrotherapy had a higher demand for epidural analgesia. Several studies [12,23,24,25,26,27] support these findings by explaining how hydrotherapy reduces stress hormones, facilitating neurohormonal interactions [28] that decrease pain perception during labor.

Hydrotherapy also implies less use of epidural analgesia according to our results. While in the hydrotherapy group, one in five women used an epidural (20%), in the group that did not use hydrotherapy, the demand for this technique was higher, with two in five women using an epidural (40%), admitting significant differences between the two groups, which associate hydrotherapy with fewer epidurals. We also found results in the literature indicating even less use of the epidural technique with water immersion, such as the study by Camargo et al. [29], where 91.1% of women felt comfortable and remained in the water, only 5.6% wanted to stop using water, and just 1.1% used epidural analgesia. In the study by Bayle et al. [1], the use of other drugs for pain relief varied from 10.2% to 18.5% between the group that used water and the group that did not use water, respectively.

This study may present some selection bias, both from the women themselves and the healthcare professionals attending the births. Regarding bias among the participant women, there may be bias due to the self-selection of hydrotherapy use during childbirth. The participant women may have been previously conditioned regarding the use of hydrotherapy during the birthing process.

Regarding healthcare professionals, bias may also exist due to their desire to promote or discourage the use of hydrotherapy, based on personal interest in its use or a lack of knowledge and training in the techniques involved.

Our study spanned over ten years, and many healthcare professionals have been involved throughout this period, so it is not possible to determine if there were any staff who promoted or discouraged the use of hydrotherapy during childbirth.

Future research should recognize the importance of including clinical trials in which sample selection considers the pain tolerance of the selected women, as well as relevant variables such as parity and prior knowledge of the technique. The inclusion of these variables could improve internal validity and data extrapolation.

## 5. Conclusions

Our data show that hydrotherapy was associated with a longer time to delivery. Additionally, hydrotherapy was also associated with a lower perception of pain and a lower frequency of requests for epidural analgesia.

## Figures and Tables

**Table 1:** Baseline characteristics of the sample.

		No Hydrotherapy		Hydrotherapy		*p*
		*n*	%	*n*	%	
Total		253	67.1	124	32.9	
Age	Mean SD	32.5 (5.3)	-	31.7 (5.6)	-	0.103
Abortions	Absence	201	67.9	95	32.1	0.62
	Presence	52	64.2	59	35.8	
Parity	0	94	55	77	45	<0.001
	1	109	73.2	40	26.8	
	≥2	50	87.7	7	12.3	
Initiation of Labor	Spontaneous	208	63.8	118	36.2	<0.001
	Induced	45	88.2	6	11.8	
Newborn Weight (grams)	Mean SD	3321.9 (446.3)	-	3306.3 (378.8)	-	0.369

## Data Availability

Data regarding this study are available upon request from the corresponding author.

## Public Involvement Statement

There was no public involvement in any aspect of this research.

## Guidelines and Standards Statement

This manuscript was drafted according to the Strengthening the Reporting of Observational Studies in Epidemiology (STROBE) approach, and the associated guidelines were followed in this observational study.
